# PD-1 inhibitors combined with paclitaxel and cisplatin in first-line treatment of esophageal squamous cell carcinoma (ESCC): a network meta-analysis

**DOI:** 10.1186/s12885-023-11715-3

**Published:** 2023-12-11

**Authors:** Jia Zhao, Simeng Zhang, Xiaoyu Guo, Ce li, Bowen Yang, Xiujuan Qu, Shuo Wang

**Affiliations:** 1 Department of Medical Oncology, The First Hospital of China Medical University, Shenyang, China; 2 Key Laboratory of Anticancer Drugs and Biotherapy of Liaoning Province, The First Hospital of China Medical University, Shenyang, China; 3 Liaoning Province Clinical Research Center for Cancer, The First Hospital of China Medical University, Shenyang, China; 4 Key Laboratory of Precision Diagnosis and Treatment of Gastrointestinal Tumors, Ministry of Education, The First Hospital of China Medical University, Shenyang, China

**Keywords:** Esophageal squamous carcinoma, Paclitaxel, Cisplatin, PD-1 inhibitor, Meta analysis

## Abstract

**Background:**

The combinations of PD-1 inhibitors with paclitaxel/cisplatinum (PD-1 + TP) and fluoropyrimidine/cisplatinum (PD-1 + FP) both have been shown to improve overall survival (OS) and progression-free survival (PFS) in patients with previously untreated, advanced esophageal squamous cell carcinoma (ESCC). However, there is no consensus on which chemotherapy regimen combined with PD-1 has better efficacy. To deal with this important issue in the first-line treatment of patients with ESCC, a network meta-analysis (NMA) was performed.

**Methods:**

Data were collected from eligible studies searched in Medline, Web of Science, PubMed, the Cochrane Library and Embase. The pooled hazard ratio (HR) for the OS, and PFS, odds ratio (OR) for the objective response rate (ORR) and ≥ 3 grade treatment-related adverse events (≥ 3TRAEs) were estimated to evaluate the efficacy of PD-1 inhibitors combined with TP or FP.

**Results:**

Five RCTs and one retrospective study involving 3685 patients and evaluating four treatments were included in this NMA. Compared to other treatments, PD-1 + TP was better. For the PFS, the HRs for PD-1 + TP compared to PD-1 + FP, TP and FP were 0.59 (0.44, 0.80), 0.56 (0.51, 0.61) and 0.45 (0.37, 0.56) respectively. For the OS, PD-1 + TP was also a better treatment compared to other treatments. The HRs were 0.74 (0.56, 0.96), 0.64 (0.57, 0.71), 0.53 (0.43, 0.67) respectively. For the ORR, there was no significant difference between PD-1 + TP and PD-1 + FP, and the ORs were 1.2 (0.69, 2.11). Compare with TP and FP, PD-1 + TP had an obvious advantage, ORs were 2.5 (2.04, 3.04) and 2.95 (1.91, 4.63). For ≥ 3TRAEs, PD-1 + TP compared to other treatments, ORs were 1.34 (0.74, 2.46) and 1.13 (0.92, 1.38) and 2.23 (1.35, 3.69).

**Conclusion:**

PD-1 + TP significantly improved both PFS and OS compared to PD-1 + FP. Taking into account both efficacy and safety, PD-1 + TP may be a superior first-line treatment option for ESCC.

**Supplementary Information:**

The online version contains supplementary material available at 10.1186/s12885-023-11715-3.

## Introduction

Esophageal cancer, with its high incidence and poor prognosis, is the seventh most common cancer and sixth leading cause of cancer death worldwide [1], with a 5-year survival rate of approximately 15%-20% [2]. Overall survival (OS) is less than one year if advanced esophageal cancer is detected [3, 4]. However, esophageal squamous cell carcinoma (ESCC) is not listed separately in major guidelines. Like adenocarcinoma, fluoropyrimidine and cisplatinum (FP) regimens are recommended as first-line treatment [5, 6]. In Asia, ESCC is a more common pathological type. Some studies have compared the efficacy of paclitaxel-based regimen and fluorouracil-based regimen in ESCC [7–10], but there is no consistent conclusion. In the era of immunotherapy, clinical studies have proved that chemotherapy combined with immune checkpoint inhibitors (PD-1 inhibitors) can confer greater benefit to patients with esophageal cancer than chemotherapy alone, especially in ESCC. For the first-line treatment of ESCC, FP or paclitaxel and cisplatin (TP) were selected as the chemotherapy regimen combined with PD1 inhibitors in different randomized controlled trials (RCTs) [4, 11–14]. However, for FP or TP, which regimen is more suitable for first-line treatment, and whether immunotherapy combined with different chemotherapy schemes has different efficacies, there is no direct comparative study. Thus, the value of pairwise meta-analysis is limited. Meta-analysis, which compares the advantages and disadvantages of multiple treatments simultaneously, is a better method of analysis [15]. Therefore, in the present study, NMA was performed to propose a better treatment plan in the first-line treatment of patients with ESCC.

## Methods

### Search strategy

We conducted a comprehensive search of Medline, Web of Science, Pubmed, the Cochrane Library and Embase databases up to April 2023. In addition, we systematically reviewed all abstracts from the American Society of Clinical Oncology (ASCO) and the European Society of Medical Oncology (ESMO) Congress between 2012 and 2023. Our search strategy was designed to identify published randomized controlled trials that evaluated first-line treatment options for patients with esophageal cancer.

### Selection criteria

All RCTs assessing first-line treatments for esophageal cancer were included in this systematic review, without any restrictions on publication date, location or language. Eligible studies had to meet the following criteria: 1) prospective phase III randomized controlled trials; 2) included patients with metastatic, unresectable, or recurrent squamous of the esophagus; 3) first-line treatment setting; 4) compared at least two arms that consisted of the following agents: FP(5-fluorouracil, cisplatin), TP (paclitaxel, cisplatin), and PD-1 inhibitors.

### Data extraction and quality assessment

Two authors (Zhao and Zhang) independently scrutinized the titles and abstracts of retrieved RCTs. They reviewed the full texts of selected RCTs to evaluate eligibility criteria for inclusion in NMA, extracted study characteristics and outcome data. Qu resolved any disagreements between the authors if necessary.

The Cochrane tools were utilized to evaluate the quality and risk of bias. HRs with their corresponding 95% CI for PFS and OS were extracted from various studies, while OR and its 95% CI were employed to indicate the frequencies of ORR. In cases where data on HR and its 95% CI could not be obtained, Kaplan–Meier curves were digitized using Engauge Digitizer (www.digitizer.sourceforge.net) followed by hazard ratio calculation in R.

### Statistical analysis

Pairwise meta-analyses were conducted using the JAGS ‘Gemtc’ package in R software (version 4.0.3) [16, 17]. Heterogeneity among studies was evaluated by means of the Q test and I^2^ statistic [18]. The fixed-effect or random-effects model was selected based on the value of I^2^ (< 50% or > 50%, respectively). Results from pairwise meta-analysis were presented as HR with 95% CI for OS and PFS, and as OR with 95% CI for ORR and ≥ 3TRAEs. Network plots were generated using R (version 4.0.3) to compare different treatments and depict network geometry. Network meta-analysis (NMA) was performed under the Bayesian framework using JAGS and the "gemtc" package in R [17]. Both random effects and consistency models were utilized in NMA, with four independent Markov chains automatically generated for posterior distribution estimation through 5000 adaptation iterations and 20,000 inference iterations per chain.Run lengths were extended if the Brooks-Gelman-Rubin diagnostic or time series plots indicated that the Markov chains had not converged (Supplementary Fig. 2). The NMA results were presented as HRs with 95% credible intervals (CIs) for OS and DFS, and ORs with 95% CIs for ORR and ≥ 3TRAEs. The probability of each treatment regarding survival outcomes was ranked according to the HRs and the posterior probabilities. Two-sided *p* < 0·05 indicates statistical significance.

Consistency, global inconsistency assessment, and local inconsistency assessment were conducted to evaluate the study. The evaluation of global inconsistency was based on comparing the fit of consistency and inconsistency models using deviance information criterion (DIC). Similar DIC values among different models indicate good consistency [19, 20]. The local inconsistency was evaluated through comparing the direct and indirect evidence generated under the Bayesian framework using the node-splitting analysis, where *p* < 0·05 indicates significant inconsistency [21].

## Results

### Description of selected trials

**Fig. 1:**
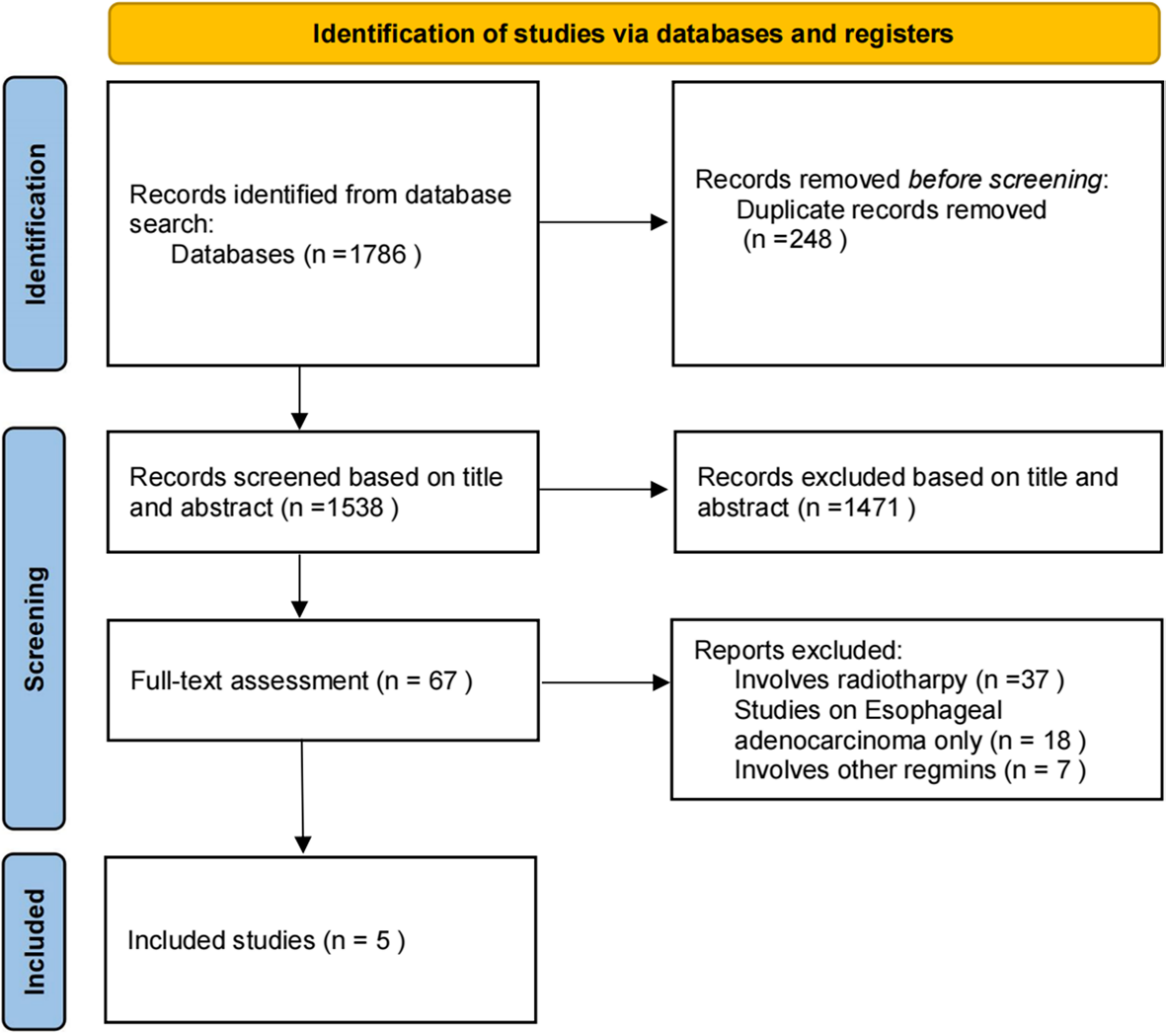
Flowchart showing the reference-selection process for meta-analysis

Eventually, after screening 1786 articles, only five RCTs met the eligibility criteria for this network meta-analysis (NMA) [4, 11–14] (Fig. 1). However, due to the inability to compare treatments simultaneously in a network among these RCTs, we identified a retrospective control study during our search that could link RCTs to a network and provide data on PFS, OS, ORR, and ≥ 3TRAEs [22]. Therefore, this study was included in our NMA. All clinical trials included in the present meta-analysis are listed in Table 1. A total of 3360 subjects were included, all of them received first-line treatment. Four treatment regimens (PD-1 + TP, PD-1 + FP, TP, FP) were included. KEYNOTE-590 and CheckMate-648 were compared with PD-1 + FP and FP. In KEYNOTE-590, the eligible patients had “unresectable or metastatic adenocarcinoma or squamous cell carcinoma of the esophagus or Siewert type 1 gastro-esophageal junction adenocarcinoma” [11]. In this NMA, only the squamous cell sub-group was included. CheckMate-648 included three arms, among which, the third arm (I + P) did not meet the standards, so was not included. The comparison between PD-1 + TP and TP was conducted via Escort1-st and JUPITER06 studies. In Orient-15, TP was administered as the base-line regimen to 94.5% of subjects. Therefore, the two arms of Orient-15 were considered PD-1 + TP and TP in this NMA. The hazard ratios (HRs) for the PFS and OS were not reported by Liu Y and Ren Z. The Kaplan–Meier curves were digitized using Engauge Digitizer (www.digitizer.sourceforge.net), and HRs were calculated in R. The OS and PFS were analyzed across all six trials, while only five trials were included for the ORR and ≥ 3TRAEs. Data on the ORR in the squamous subgroup were not provided in Keynote590.

**Table 1:** Baseline characteristics of studies included in the NMA. PD-1: programmed death receptor 1 inhibitors; TP: Paclitaxel plus cis-platinum; FP: fluorouracil plus cis-platinum

Study(year)	Author	Randomized patients	Histology	Regimen	Area
KEYNOTE 590 (2019)	Sun JM	274	squamous	PD1 + FP	GLOBLE
		274	squamous	FP	GLOBLE
ESCORT-1st (2021)	Luo H	298	squamous	PD1 + TP	CHINA
		298	squamous	TP	CHINA
CHECKMATE 648 (2022)	Doki Y	321	squamous	PD1 + FP	GLOBLE
		324	squamous	FP	GLOBLE
JUPITER 06 (2022)	Wang ZX	257	squamous	PD1 + TP	CHINA
		257	squamous	TP	CHINA
ORIENT 15 (2022)	Lu Z	327	squamous	PD1 + TP	GLOBLE
		332	squamous	TP	GLOBLE
TP vs FP (2016)	Liu Y	195	squamous	TP	CHINA
		203	squamous	FP	CHINA

### Risk of bias and heterogeneity assessment

The primary outcome was the OS, while secondary endpoints included the PFS, ORR and ≥ 3TRAEs. Study quality was assessed using the Cochrane Risk of Bias tool, version 5.1.0, with items scored as low, high or unknown risk of bias. Based on *I*^2^ < 50%, there was no significant heterogeneity observed among the trials in the network.

### Results of NMA and ranking of treatments

**Fig. 2:**
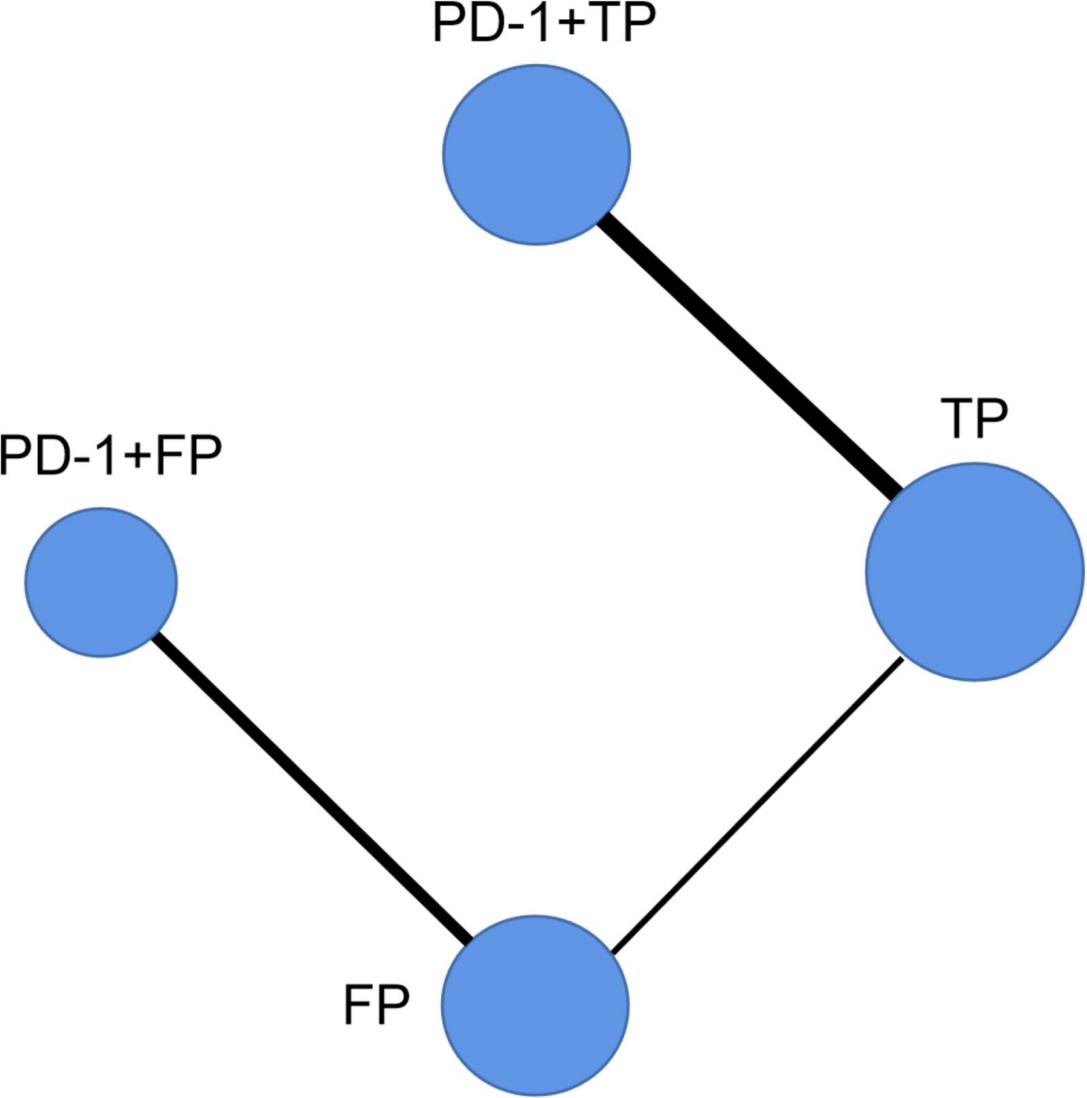
Network of all treatment comparisons for the OS and PFS. The lines connect the regimens that were directly compared in clinical trails. The thickness of the lines corresponds to the number of RCTs. PD-1: Programmed death receptor 1 inhibitors; TP: Paclitaxel plus cis-platinum; FP: fluorouracil plus cis-platinum

**Fig. 3:**
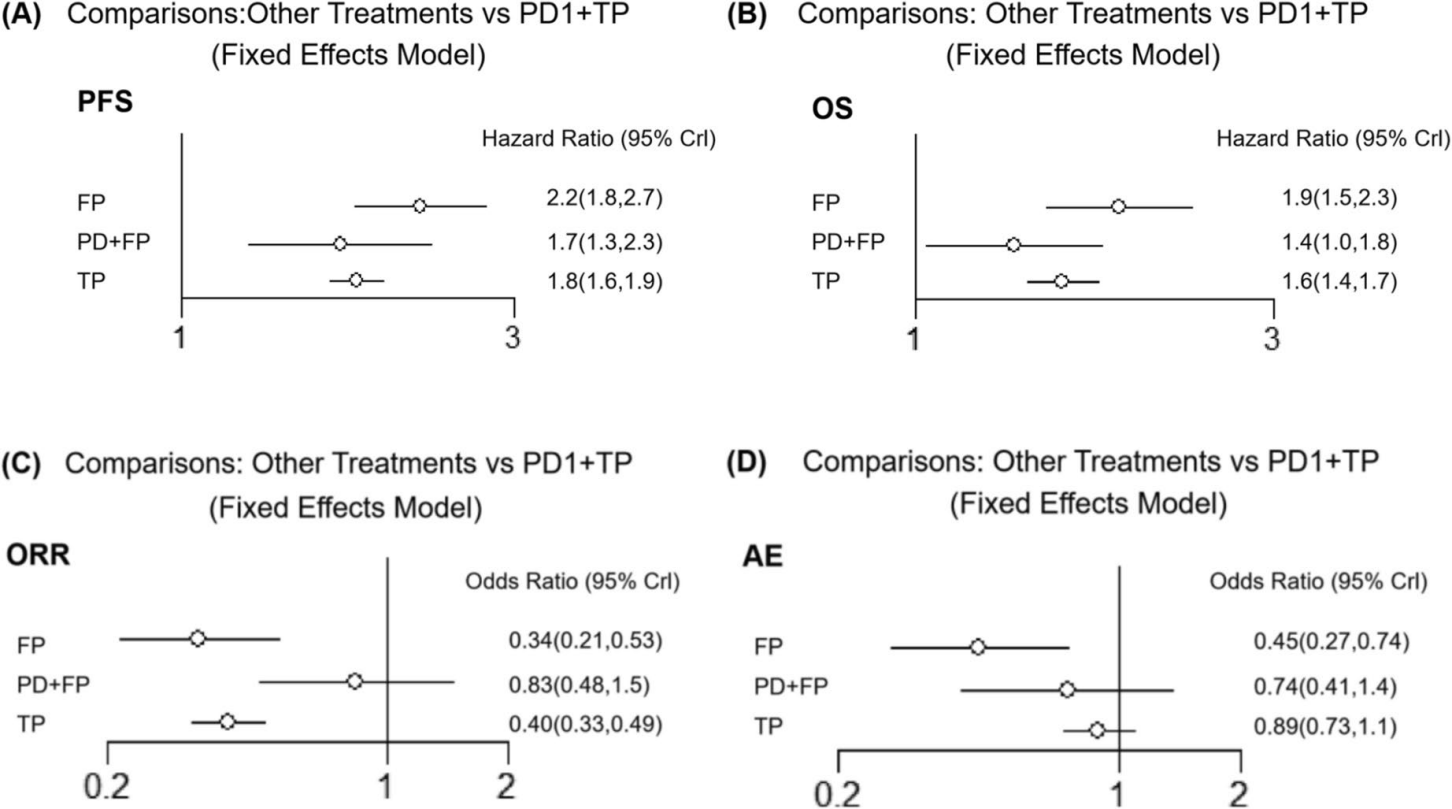
Forest plots of all individual regimens compared with PD1 + TP. HRs and 95% credible intervals are given. **A** PFS of all individual regimens compared with PD1 + TP; **B** OSs of all individual regimens compared with PD1 + TP; **C** ORRs of all individual regimens compared with PD1 + TP; **D** ≥ 3TRAEs of all individual regimens compared with PD1 + TP

A network consisting of four treatments, namely PD-1 + TP, PD-1 + FP, TP, and FP (Fig. 2), was established. The results of the comparison among all treatments are summarized in Table 2. Given that there was no statistical heterogeneity in this network (*I*^2^ = 0%), a fixed effects model was adopted to report the findings. In terms of PFS, PD-1 + TP demonstrated superior efficacy compared with other treatments. The HRs for PD-1 + TP compared to PD-1 + FP, TP and FP were 0.59 (95% confidence interval [CI]: 0.44–0.80), 0.56 (95% CI: 0.51–0.61), and 0.45 (95% CI: 0.37–0.56), respectively (Table 2A). In terms of the OS, PD-1 + TP exhibited superior efficacy compared to other treatments. The HRs for PD-1 + TP compared to PD-1 + FP, TP and FP were 0.74 (95% CI: 0.56, 0.96), 0.64 (95% CI: 0.57, 0.71), 0.53 (95% CI: 0.43, 0.67) respectively (Table 2A). Due to the lack of data on the ORR in the squamous subgroup in Keynote590, only five trials were included for analysis of the ORR and ≥ 3TRAEs (Supplementary Fig. 1). No significant difference in the ORR was observed between PD-1 + TP and PD-1 + FP, as indicated by RRs of 1.2 (95% CI:0.69, 2.11). However, PD-1 + TP showed a significant advantage over TP and FP with ORs of 2.5 (95% CI: 2.04, 3.04) and 2.95 (95% CI: 1.91, 4.63), respectively (Table 2B). For security, PD-1 + TP had no significant difference with PD-1 + FP and TP, ORs of ≥ 3TRAEs were 0.75(95% CI:0.41,1.4) and 0.89(95% CI:0.73, 1.1). The results suggest that FP may have had superior security, with an OR of 2.23 (1.35, 3.69) (Table 2B). Forest plots were generated to visualize the network estimates (Fig. 3).

**Table 2:**
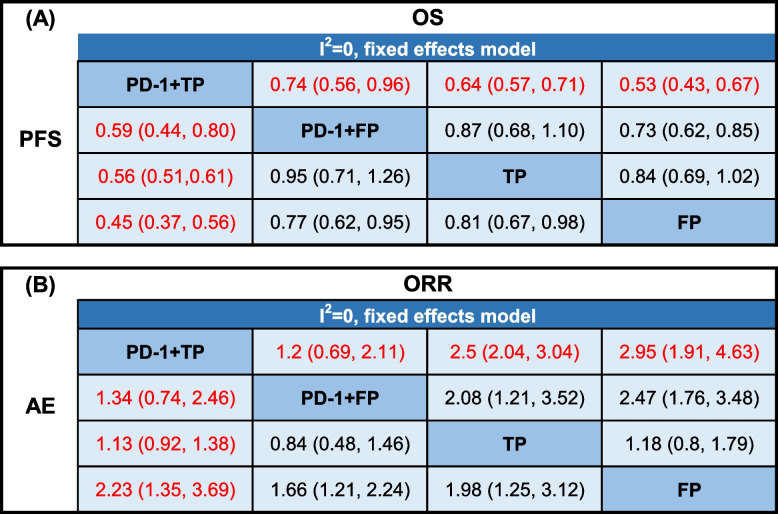
Results of the network meta-analysis (NMA) comparing four treatment regimens simultaneously in terms of the OS, progression-free survival (PFS), objective response rate (ORR) and ≥ 3 grade treatment-related adverse events (≥ 3 TRAEs) are presented. (A) The relative effects, expressed as hazard ratios (HRs) with 95% confidence intervals, are shown for PD-1 + TP; these results demonstrate superiority compared to PD-1 + FP in terms of the PFS and OS. The HRs for a given comparison can be found at the intersection of two treatments. (B) The relative effects, expressed as odds ratios (ORs) with 95% confidence intervals, are presented for PD-1 + TP; in comparison to PD-1 + FP, there is no discernible advantage of PD-1 + TP in terms of the ORR and safety. The ORs for a given comparison can be found at the intersection of two treatments

## Discussion

Causes of distinct variations in the risk factors, incidence and distribution worldwide between both histological types of esophageal cancer are multifaceted [23]. Extensive clinical trials in western nations have primarily focused on patients with adenocarcinoma of the esophagus, and fluorouracil-based chemotherapy has been recommended for esophageal adenocarcinoma [24]. With the advancement of research of esophageal cancer, it has become evident that ESCC and adenocarcinoma are two distinct diseases. Currently, more clinical trials are focusing on ESCC, particularly in the “esophageal cancer belt”, which includes parts of northern Iran, southern Russia, central Asian countries, and northern China where squamous cell cancers account for up to 90% of all cases [25]. Paclitaxel combined with cisplatin is a widely used therapeutic regimen for ESCC in China. In a clinical investigation by Zhang L., the combination of paclitaxel and platinum showed improved survival rates during the postoperative adjuvant treatment of ESCC. Compared with the control group, the 3-year DFS rates were 56.3% *v*. 34.6% (*P* = 0.006). The 3-year OS rates were 55.0% *v*. 37.5% (*P* = 0.013) [26]. In a study by Kim J.Y., patients with advanced ESCC were treated with paclitaxel plus platinum. The median PFS was 5.0 months and median survival was 8.3 months, and the objective tumor response rate was 33.3%. TP is a standard chemotherapy regimen for first-line treatment of ESCC. In other types of squamous cell carcinomas, such as head and neck or lung cancer, anti-microtubule drugs are also recommended as a priority [27].

In the era of immunotherapy, PD-1 combined chemotherapy has demonstrated superior efficacy compared to traditional chemotherapy in various tumors, particularly non-small-cell lung cancer [28]. Recently, the use of immunotherapy in esophageal cancer has been increasingly promoted from second-line to first-line treatment. Significant benefits were observed with PD-1 + FP compared to FP in both KEYNOTE-590 and CheckMate-648 trials. In the KEYNOTE-590 trial, pembrolizumab plus chemotherapy exhibited a superior OS of 12.4 months compared to placebo-chemotherapy with an OS of 9.8 months (*p* < 0.0001) among all randomized patients; in the ESCC subgroup, the OS was also significantly improved at 12.6 months *v.* 9.8 months (*p* = 0.0006). Additionally, the PFS was prolonged from 5.8 months to 6.3 months (*p* = 0.0001).

In the CheckMate-648 study, the overall population demonstrated a significantly longer OS with nivolumab plus FP compared to FP alone (13.2 months *v*. 10.7 months; *P* = 0.002). Three additional RCTs, ESCORT1st, JUPITER06, and ORIENT05, initiated by Chinese scholars also confirmed that PD-1 combined with chemotherapy provided greater benefits than chemotherapy alone in first-line treatment of ESCC. The results of all three studies proved that the benefits of combining PD-1 with chemotherapy were significantly greater than those of chemotherapy alone, as evidenced by an OS rate of 15.3 months *v*. 12 months, 17 months *v*. 11 months, and 16.7 months *v*. 12.5 months respectively. Furthermore, the safety profile was comparable.

Immunotherapy combined with chemotherapy is recommended as first-line treatment of ESCC by the aforementioned RCTs. Notably, in studies comparing PD-1 + TP to TP (ESCORT1st, JUPITER06, ORIENT 05), the experimental groups demonstrated an OS of 15–17 months; however, in RCTs comparing PD-1 + FP to FP (KEYNOTE-590 and CheckMate-648), the experimental groups exhibited an OS ranging from 10–13 months. Could the differential effect be attributed to the combination of immunotherapy with different chemotherapy regimens? As there has been no head-to-head comparison between PD-1 + TP and PD-1 + FP, we designed a NMA to compare which chemotherapy combined with PD-1 is more beneficial in the first-line treatment of ESCC.

In this NMA, PD-1 + TP exhibited improved survival compared to PD-1 + FP. Previous studies have suggested a stronger synergistic effect between paclitaxel and immunotherapy. The mechanism can be explained by the fact that paclitaxel exhibits a stronger ability to enhance immunogenicity of cell death, thereby shaping a more favorable inflammatory immune microenvironment and promoting activation of immune cells through release of various proinflammatory cytokines by tumor cells; when combined with PD-1, it may achieve better synergistic effects [29–32].

The primary indications of esophageal cancer patients are progressive dysphagia and retrosternal pain, with malnutrition frequently resulting from the former and negatively impacting prognosis [33]. Achieving superior disease control rates is particularly crucial in treating this condition. In ESCORT-1st, the ORR for PD-1 + TP was 72.1%, while CheckMate-648 saw an ORR of 47% for PD-1 + FP. In terms of numerical values, there was a significant gap between the two regimens; however, this NMA found no statistical difference in ORRs between PD-1 + TP and PD-1 + FP, which may be attributed to the limited number of studies included in the analysis of ORR. As a potentially superior alternative, PD-1 + TP has been extensively investigated in neoadjuvant treatments for ESCC, including KEYSTONE-001 [34] (MPR 72.4%) and ESPRIT (ORR 66.67%) [35]. These studies have demonstrated high response rates with the use of PD-1 + TP. However, further RCTs are necessary to provide more compelling evidence.

Limitations: some limitations of this NMA should be acknowledged. In the five RCTs involved in this NMA, the group of ESCC with highly expressed PD-L1 had a more benefit from PD-1 plus chemotherapy, but subgroups defined were different. Some limitations of this NMA must be acknowledged: although the group with high PD-L1 expression in ESCC benefited more from PD-1 plus chemotherapy in the five RCTs included, but in this NMA, we found that there were differences in subgroup definitions. CheckMate-648 defined subgroups according to the tumor cell PD-L1 expression of 1% or greater, but KEYNOTE-590 and ORIENT-15 subgroups were defined by CPS ≥ 10. In ESCORT-1st analysis the three different selected PD-L1 expressions were classified into subgroups (TPS ≥ 10, TPS ≥ 1, TPS < 1): regardless of the PD-L1 expression level, patients can benefit from combination immunotherapy. JUPITER 06 did not set subgroups for different PD-L1 expressions, but the analysis incorporated all randomized patients *in toto*. Therefore, this MNA could not perform subgroup analysis based on the level of expression of PD-L1.

## Conclusion

Based on the findings of this NMA, it appears that the combination therapy of TP and PD-1 inhibitors is a superior first-line treatment option for ESCC. Further RCTs are warranted to optimize first-line treatment strategies and generate evidence-based medicine.

### Supplementary Information

**Additional file 1: Data 1.** Literature search strategies on PubMed. **Supplementary Table 1.** Checklist of the PRISMA extension for network meta-analysis. **Supplementary Figure 1.** Network of alltreatment comparisons for ORR and ≥3TRAEs. The lines connect the regimens that were directly compared in clinical trails. The thickness of the lines corresponds to the number of RCTs. PD1:Programmed death receptor 1 inhibitors; TP: Paclitaxelpluscis-platinum; FP:fluorouracilpluscis-platinum. **Supplementary Figure 2.** Convergence of the four chains established by inspection of the Brooks-Gelman-Rubin diagnostic and the density trace plot. (A) PFS; (B) OS; (C) ORR; (D) ≥3TRAEs.

## Data Availability

The data that support the findings of this study are available from the corresponding author upon reasonable request.

## Abbreviations

NMA: Network Meta-analysis
ESCC: Esophageal Squamous Cell Carcinoma
RCTs: Randomized Controlled Trials
TRAEs: Treatment-related Adverse Events
OS: Overall Survival
PFS: Progression-free Survival
HR: Hazard Ratio
ORR: Objective Response Rate
DIC: Deviance Information Criterion
PD-1 + TP: PD-1 Inhibitors with Paclitaxel/Cisplatinum
PD-1 + FP: PD-1 Inhibitors with Fluoropyrimidine/Cisplatinum
FP: Fluoropyrimidine and Cisplatinum
TP: Paclitaxel and Cisplatinum
